# EGFR wild-type amplification and activation promote invasion and development of glioblastoma independent of angiogenesis

**DOI:** 10.1007/s00401-013-1101-1

**Published:** 2013-02-22

**Authors:** Krishna M. Talasila, Anke Soentgerath, Philipp Euskirchen, Gro V. Rosland, Jian Wang, Peter C. Huszthy, Lars Prestegarden, Kai Ove Skaftnesmo, Per Øystein Sakariassen, Eskil Eskilsson, Daniel Stieber, Olivier Keunen, Narve Brekka, Ingrid Moen, Janice M. Nigro, Olav K. Vintermyr, Morten Lund-Johansen, Simone Niclou, Sverre J. Mørk, Per Øyvind Enger, Rolf Bjerkvig, Hrvoje Miletic

**Affiliations:** 1 Department of Biomedicine, University of Bergen, Jonas Lies vei 91, 5009 Bergen, Norway; 2 Department of Neurosurgery, Hospital Cologne Merheim, 51109 Cologne, Germany; 3 Department of Dermatology, Haukeland University Hospital, 5021 Bergen, Norway; 4 NorLux Neuro-Oncology Laboratory, CRP-Santé, 1526 Luxembourg, Luxembourg; 5 Department of Pathology, The Gade Institute, Haukeland University Hospital, Jonas Lies vei 65, 5021 Bergen, Norway; 6 Department of Neurosurgery, Haukeland University Hospital, 5021 Bergen, Norway; 7 Institute of Surgical Science, University of Bergen, Jonas Lies vei 91, 5009 Bergen, Norway

**Keywords:** Glioblastoma, EGFR amplification, Invasion

## Abstract

Angiogenesis is regarded as a hallmark of cancer progression and it has been postulated that solid tumor growth depends on angiogenesis. At present, however, it is clear that tumor cell invasion can occur without angiogenesis, a phenomenon that is particularly evident by the infiltrative growth of malignant brain tumors, such as glioblastomas (GBMs). In these tumors, amplification or overexpression of wild-type (wt) or truncated and constitutively activated epidermal growth factor receptor (EGFR) are regarded as important events in GBM development, where the complex downstream signaling events have been implicated in tumor cell invasion, angiogenesis and proliferation. Here, we show that amplification and in particular activation of wild-type EGFR represents an underlying mechanism for non-angiogenic, invasive tumor growth. Using a clinically relevant human GBM xenograft model, we show that tumor cells with EGFR gene amplification and activation diffusely infiltrate normal brain tissue independent of angiogenesis and that transient inhibition of EGFR activity by cetuximab inhibits the invasive tumor growth. Moreover, stable, long-term expression of a dominant-negative EGFR leads to a mesenchymal to epithelial-like transition and induction of angiogenic tumor growth. Analysis of human GBM biopsies confirmed that EGFR activation correlated with invasive/non-angiogenic tumor growth. In conclusion, our results indicate that activation of wild-type EGFR promotes invasion and glioblastoma development independent of angiogenesis, whereas loss of its activity results in angiogenic tumor growth.

**Electronic supplementary material:**

The online version of this article (doi:10.1007/s00401-013-1101-1) contains supplementary material, which is available to authorized users.

## Introduction

Human glioblastoma (GBM) is the most frequent and most malignant primary brain tumor. The majority of GBMs arise de novo and are defined as primary GBMs, while the progression from lower grade astrocytomas results in secondary GBMs [34]. Primary GBMs most frequently harbor the common mutations 9p and 10q loss as well as amplification of epidermal growth factor receptor (*EGFR*), a tyrosine kinase receptor [34]. Wild-type (wt) *EGFR* is amplified in 40–50 % of primary GBMs and a fraction of *EGFR*-amplified tumors in addition express the mutant variant EGFRvIII, a constitutively active receptor [60]. Signaling through the EGFR pathway is a complex process that involves tight regulation of several intracellular cell signaling networks [10]. When these regulatory networks are altered, as in cancer, they have been shown to contribute to malignant transformation and tumor progression through increased cell proliferation, angiogenesis, invasion, and metastasis [23, 37, 39, 43].

The diffuse infiltrative growth of tumor cells within the central nervous system (CNS) still represents a major problem for effective therapeutic intervention as the delivery of active therapeutic agents to the invasive tumor cells is limited by the blood–brain barrier (BBB). While factors that mediate tumor angiogenesis have been well defined [7, 25, 51, 56], the major mechanisms causing non-angiogenic, invasive tumor growth in vivo still remain elusive. This can partly be explained by the lack of representative animal models that reflect the invasive tumor growth seen in patients. To this end, we and others have shown that human GBMs, short-term cultured as multicellular biopsy spheroids, maintain the same DNA copy number as the parental tumors [14] and can, when xenotransplanted into the CNS of immunodeficient rats, grow invasively for extensive periods without switching to angiogenic tumor growth [53, 59]. Thus, in our model system there appears to be a selection toward a subpopulation of glioma cells, which is capable of initiating and sustaining tumor growth independent of angiogenesis. In the present study, we show that wtEGFR activation is associated with non-angiogenic, infiltrative tumor development both in our animal model as well as human GBMs and that inactivation of the receptor can lead to angiogenic tumor growth.

## Materials and methods

### Cell culture

Biopsy spheroids were prepared as described previously [5]. After 1–2 weeks in culture, spheroids with diameters between 200 and 300 μm were selected for intracerebral implantation. For functional experiments with cetuximab and EGFR-CD533, spheroids with a standardized cell number were generated as described under “Lentiviral EGFR-CD533 production and infection of glioblastoma cells”.

The human embryonic kidney cell line 293T (ATCC number CRL-11268) and the U87 cell line were obtained from the American Type Culture Collection (ATCC, Manassas, VA) and maintained in Dulbecco’s modified eagle medium (DMEM) supplemented with 10 % fetal calf serum (FCS) and 1 % glutamine. All cell lines were grown at 37 °C in a humidified atmosphere of 5 % CO_2_.

### In vivo experiments

Nude immunodeficient rats (rnu/rnu Rowett) were fed a standard pellet diet and were provided with water ad libitum. All procedures were approved by the Norwegian National Animal Research Authority. Biopsy spheroids were stereotactically implanted into the right brain hemisphere as described previously [53]. Rats were euthanized with CO_2_, perfused intracardially with 0.9 % NaCl and killed when symptoms developed.

Intracranial convection-enhanced delivery (CED) of cetuximab was started 6 weeks after tumor implantation and was given for 4 weeks. CED was performed using osmotic minipumps (Alzet mini-osmotic pump, model 2ML4, Durect Corp., Cupertino, CA), which maintain a constant flow of 2.5 μl/h over 28 days. Pumps were filled with the antibody at a concentration of 5 mg/ml, consequently the rats received 300 μg of the antibody per day. The pumps were connected to an intracranial catheter (Alzet Brain Infusion Kit 2). Pumps were placed subcutaneously at the back of the rats. The catheter tip was inserted through the same burr hole that had been created to inject the tumor cells and was placed approximately at the injection site of tumor cells. Control groups for cetuximab received pumps loaded with PBS.

For pimonidazole analysis, animals were injected with hypoxyprobe-1 (HPI, Burlington, MA) 30 min prior to euthanasia. Brains were removed and fixed in 4 % formalin for 1–7 days, or tumors were excised and snap frozen in liquid nitrogen for protein isolation.

### Immunohistochemistry

Immunohistochemistry of paraffin sections was performed as described previously [27]. The following primary antibodies were used: anti-human nestin diluted 1:200 (Millipore, Billerica, MA), anti-human sox2 diluted 1:200 (R&D Systems, Minneapolis, MN), anti-CD31 diluted (Santa Cruz, Santa Cruz, CA), anti-wtEGFR diluted 1:500 (Santa Cruz), anti-pEGFR (Tyr1173) diluted 1:250 (Cell Signaling, Danvers, MA), anti-pimonidazole diluted 1:200 (HPI), anti-vWF, diluted 1:500 (DAKO), anti-angiopoietin2 diluted 1:200 (Santa Cruz), anti-EGFRvIII diluted 1:200 (clone L8A, a gift kindly provided by S. Clayton, Duke University, Durham, NC) and anti-GFP diluted 1:200 (Millipore). The H&E and immunohistochemical stainings were analyzed on a Nikon light microscope (Nikon, Tokyo, Japan) using Nikon imaging software. The quantification of vessel area fractions was performed using the Nikon imaging software. Overview pictures of histological slides were taken using a digital slide scanner and Imagescope software (Aperio, Vista, CA).

### Western blotting

Protein extraction and western blotting were performed as described previously [53]. Primary antibodies used were anti-pAkt (Ser-473) diluted 1:500 (Cell Signaling), anti-pStat3 (Tyr-705) diluted 1:2,000 (Cell Signaling), anti-pMAPK (Thr-202/Tyr-204) diluted 1:2,000 (Cell Signaling), anti-EGFR diluted 1:500 [Life Technologies (Biosource)], anti-EGFRvIII diluted 1:1,000 (clone L8A, a gift kindly provided by S. Clayton, Duke University, Durham, NC), anti-VEGF diluted 1:200 (Santa Cruz), anti-HIF-1α diluted 1:500 (Becton–Dickinson, San Jose, CA), anti-angiopoietin1 diluted 1:300 (Santa Cruz), anti-angiopoietin2 diluted 1:500 (Santa Cruz), anti-FGF2 diluted 1:500 (Santa Cruz), anti-CD133/1 clone AC133 diluted 1:100 (Miltenyi, Bergisch-Gladbach, Germany), anti-vimentin diluted 1:500 (DAKO), anti-snail diluted 1:100 (Abgent, San Diego, CA), anti-Twist diluted 1:100 (Santa Cruz), anti-beta-Actin diluted 1:1,000 (Abcam, Cambridge, UK) and anti-GAPDH diluted 1:2,500 (Abcam).

The primary antibody was detected using a goat F(ab′)2 fragment anti-rabbit IgG (H + L)-peroxidase diluted 1:100,000 (Beckman Coulter, Brea, CA), or goat anti mouse IgG-HRP diluted 1:25,000 (Santa Cruz) or HRP-conjugated goat anti-rabbit/mouse secondary antibody (Immunotech, Fullerton, CA) diluted 1:2,500.

### Cloning of EGFR-CD533

The EGFR-CD533 construct was a gift from Joseph Contessa, Yale University School of Medicine, New Haven, CT. From this plasmid, EGFR-CD533 was amplified by PCR using 5′-GCATCATCTAGAGCCACCATGCGACCCTCCGGG-3′ as forward and 5′-GCATCACTCGAGTCAGCGCTTCCGAACGATG-3 as reverse primer. The primers were designed to insert XbaI and XhoI restriction sites flanking the EGFR-CD533 gene. The lentiviral vector pRRL.sinCMVeGFPpre [47] was cut with XbaI and SalI to remove the eGFP gene. The PCR product was cut with XbaI and XhoI and ligated into the lentiviral vector.

### Lentiviral EGFR-CD533 production and infection of glioblastoma cells

Lentiviral vectors carrying EGFR-CD533 or GFP were produced in 293T cells using FuGene HD transfection reagent (Life technologies, Paisley, UK) according to the manufacturer’s instructions. The production and titration of lentiviral vectors were performed as described previously [18]. For infection, spheroids were dissociated using the Neuronal dissociation kit (Miltenyi, Bergisch-Gladbach, Germany), plated in round-bottomed 96 wells with 3,000 cells/well in culture medium with 4 % methylcellulose, and infected with viral supernatants at an MOI of 5–30. 96-well plates were centrifuged for 1.5 h at 31 °C. Medium was changed 2 days after infection. 6 days after infection, reformed spheroids were stereotactically implanted using 10 spheroids/rat.

### Array CGH

Array CGH was performed as previously described [53].

### Gene expression analysis

RNA was purified from tissue samples using Ambion Tri-reagent (life technologies) following the manufacturer’s instructions. RNA samples were then DNAse treated using Ambions turbo DNA Free kit to remove any contaminating genomic DNA. Microarray analysis of EGFR-CD533 and control animals were carried out as specified in [50].

### Functional analysis of gene expression data

Data were analyzed using IPA (Ingenuity Systems, http://www.ingenuity.com). Right-tailed Fisher’s exact test was used to calculate a *p* value determining the probability that each biological function assigned to that data set is due to chance alone. *Upstream regulator analysis* was based on prior knowledge of expected effects between transcriptional regulators and their target genes stored in the Ingenuity^®^ Knowledge Base. Two statistical measures, standard in IPA, were used to detect potential transcriptional regulators: an overlap *P* value and an activation *z* score. First, the analysis examined how many known targets of each transcriptional regulator were present in our data set, resulting in an estimation of an overlap *P* value. We set a threshold of an overlap *P* value <0.05 to identify significant upstream regulators. Second, the known effect (activation or suppression) of a transcriptional regulator on each target gene was compared with observed changes in gene expression. Based on concordance between them, an activation *z* score was assigned, showing whether a potential transcriptional regulator was in “activated” (*z* score > 2), “inhibited” (*z* score < −2) or uncertain state.

### Fluorescence in situ hybridization (FISH)

FISH analyses of paraffin sections were performed with the Vysis LSI EGFR SpectrumOrange/CEP 7 SpectrumGreen probe (Abbott Molecular, Des Plaines, IL) using the DAKO Histology FISH Accessory Kit (DAKO, Glostrup, Denmark).

### Magnetic resonance imaging (MRI)

Axial T1-weighted (T1w) RARE sequences and (T2w) RARE sequences were acquired as described previously [58]. Tumor volumes were calculated using a volumetric approach, where masks were created in Bruker’s Paravision 5.0 software, by delineating tumor in consecutive sections of the T2-weighted images. A region growing algorithm was used to assist in finding the contours of the tumor, where the seed point was placed centrally in the tumor, and the parameters of the algorithm were optimized to include all hyperintense pixels from the tumor area.

### Tissue microarray

Paraffin sections from a tissue microarray of 243 GBM patients were prepared for H&E, immunostaining and FISH. 206 cases had sufficient material left for analyses of all markers. Scoring of stained sections was performed independently by two certified neuropathologists (SJM and HM). Scoring scheme:

1. Proportion of positive tumor cells (P): 0 % (0); 1–10 % (1); 11–50 % (2); >50 % (3)
2. Intensity of staining (I): negative (0); weak (1); moderate (2); strong (3)
3. Staining index (SI): Proportion (P) × intensity (I)

The mean SI was assessed from both scorings and served as the final score displayed in the results section.

The Norwegian Data Inspectorate and the Regional Committee for Ethics in Research have approved this project. The study was performed in accordance with the Helsinki Declaration.

### Statistical analysis

Survival was analyzed by a log-rank test based on the Kaplan–Meier test using SPSS software. Differences between pairs of groups were determined by the Student’s *t* test. *P* values <0.05 were considered significant.

## Results

### Two human GBM xenograft phenotypes can be defined by different angiogenic and invasive growth properties

**Fig. 1:**
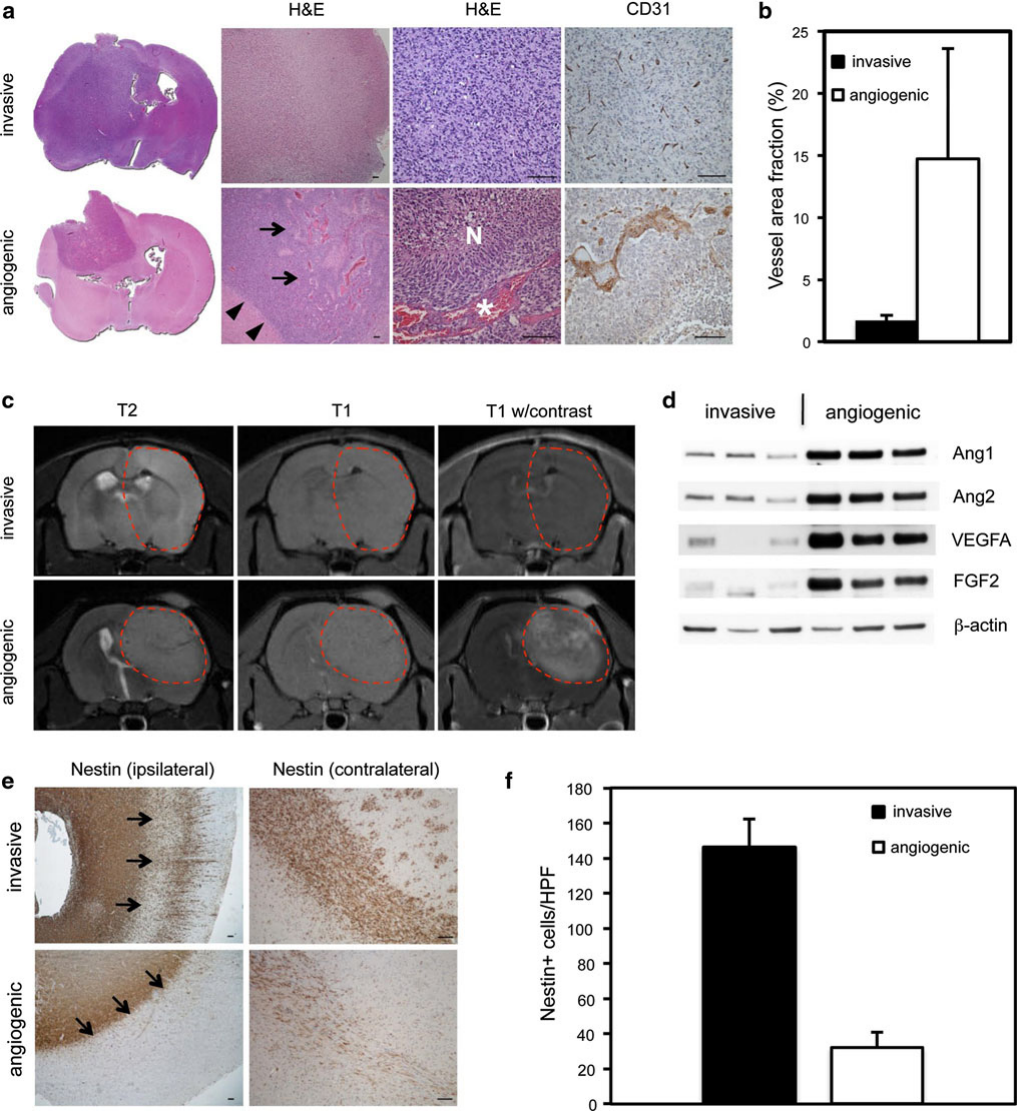
Characterization of invasive, non-angiogenic and angiogenic human glioblastoma xenografts. **a** Hematoxylin and eosin (H&E) and immunohistochemical staining of sections from invasive (P17) and angiogenic (P13) xenograft tumors with antibodies against CD31, an endothelial marker. *Arrows* point to an angiogenic area. *Arrowheads* show the sharp demarcation of angiogenic tumors. *N* depicts a necrotic area and the *asterisk* indicates microvascular proliferation. **b** Area fraction of vascular elements immunostained with vWF in invasive versus angiogenic tumors from two different animals in each group. Quantification was performed at ×200 magnification. *P* < 0.001; *n* = 20. **c** T2- and T1-weighted MRIs with and without contrast show demarcated tumors with contrast enhancement in the angiogenic group (P13), while invasive tumors (P6) have ill-defined borders and no contrast enhancement. **d** Western blot shows high expression of angiogenic factors in angiogenic (P6, P8, P22) compared to invasive tumors (P1, P3, P13). **e** Immunohistochemical staining of sections from both groups with antibodies against nestin. *Arrows* point at the white matter/cortex border demonstrating less invasion into the cortex by angiogenic tumors. **f** Quantification of invasive cells in cortical areas from two different animals in each group. *HPF* high microscopic view field (×400 magnification). *P* < 0.001; *n* = 10. Values represent mean ± SD. *Scale bars* 100 μm

We rigorously characterized the histological features of intracerebral xenografts established from 12 different patients with primary GBMs. Two distinct tumor subtypes were identified based on their ability to induce angiogenesis. As described previously, tumors in the first subtype were highly invasive and showed no signs of angiogenesis (Fig. 1a, Fig. S1) [53]. In the second subtype, tumors displayed angiogenic growth characterized by dilated macrovessels and endothelial hyperplasia. In addition, the majority of tumors also displayed typical microvascular proliferations and/or pseudopalisading necrotic areas, which are angiogenic hallmarks of GBM growth (Fig. 1a, Fig. S1, Table S1). Quantification of tumor vessels using the vascular marker vWF revealed that the area fraction of vascular elements was significantly higher in the angiogenic phenotype compared to its non-angiogenic counterpart (Fig. 1b). The angiogenic and invasive phenotypes were also verified by MRI. MRI is routinely used in the clinical setting to distinguish angiogenic (glioblastoma) from non-angiogenic tumors (low grade gliomas). Contrast enhancement represents vascular permeability and is only seen in highly angiogenic tumors [3]. In our model, the angiogenic phenotype showed more demarcated tumors and contrast enhancement on MRI, while the invasive tumors showed ill-defined borders and no contrast enhancement (Fig. 1c). To further compare the angiogenic and invasive phenotypes, we assessed expression of the pro-angiogenic factors VEGFA, ANGPT1, ANGPT2 and bFGF. As shown in Fig. 1d, the pro-angiogenic factors were mainly associated with the angiogenic phenotypes.

When comparing the invasive capabilities of both tumor subtypes, we found that the non-angiogenic phenotype showed extensive single cell infiltration into cortical brain areas in addition to migration into the contralateral hemisphere while tumor growth in the angiogenic phenotype was largely confined to the white matter with a more defined border and significantly fewer cells invading into the cortex (Fig. 1e, f).

Since various stem cell markers have been associated with a tumorigenic phenotype in human gliomas [30], we analyzed invasive and angiogenic xenografts for the expression of the stem cell markers nestin, sox2 and CD133. All tumors of either phenotype showed expression of both nestin and sox2. However, CD133, a controversial marker for stem-like cancer cells, was expressed at low to intermediate levels in two out of three xenografts of either phenotype (Fig. S2). Thus, the expression of stem cell markers was not linked to a specific phenotype.

### Amplification and activation of wild-type EGFR is associated with non-angiogenic, invasive tumor growth

**Fig. 2:**
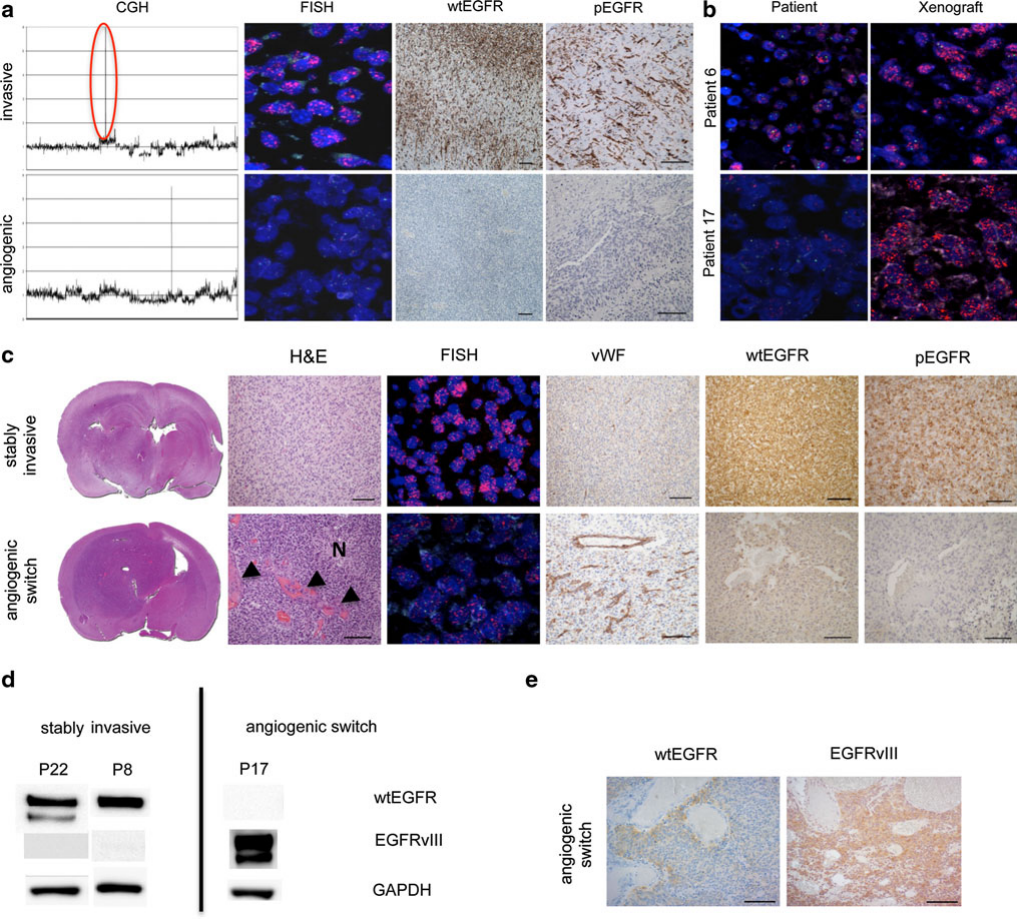
EGFR amplification and activation promote non-angiogenic tumor growth in glioblastoma xenografts. **a** aCGH of invasive (P17) and angiogenic (P13) tumors. *Red circle* highlights *EGFR* amplification. FISH with an *EGFR*/chromosome 7 probe in red and green, respectively and immunohistochemical staining of sections from invasive and angiogenic tumors with antibodies against wtEGFR and phosphorylated EGFR. Amplification, expression and activation of wtEGFR are present only in the invasive phenotype. **b** FISH with an *EGFR*/chromosome 7 probe in red and green, respectively (patient/xenograft labels correspond to Table 1). The majority of tumor cells in xenograft tumors show high *EGFR* amplification. In contrast, tumor cells from patient biopsies show variable amounts of *EGFR* amplification. **c** In vivo passaged EGFR-amplified tumor that stays stably invasive (patient 8) shows also stable wtEGFR and pEGFR expression. In contrast, an in vivo passaged EGFR-amplified tumor that switches to angiogenesis (P17) shows downregulation of wtEGFR and pEGFR in the angiogenic center. *N* depicts a necrotic area and the *arrowheads* indicate microvascular proliferation. All pictures are taken from the tumor center. *Scale bars* 100 μm. **d** wtEGFR and EGFRvIII western blot of stably invasive xenografts and xenografts that switch to angiogenesis (xenograft labels correspond to Table 1). EGFRvIII expression is lost in stably invasive xenografts, while it is upregulated upon the angiogenic switch. **e** Immunhistochemical staining with antibodies against wtEGFR and EGFRvIII. EGFRvIII is expressed in a xenograft (A3) that switches to angiogenesis, while wtEGFR is downregulated. *Scale bars* 100 μm

To determine whether angiogenic and non-angiogenic tumors differed at the molecular level, we performed array comparative genomic hybridization (aCGH). While this analysis revealed several common genomic changes in both groups, such as losses of chromosomes 9p and 10q, amplification of *EGFR* occurred exclusively in the non-angiogenic, invasive phenotype (Fig. 2a; Table 1). FISH verified these results (Fig. 2a; Table 1), but also revealed some interesting patterns regarding the presence of *EGFR* amplification. High *EGFR* amplification was detected in the tumor cells from the invasive xenografts while corresponding patient tumors showed variable levels of *EGFR* amplification in the tissue sections (Fig. 2 b). In our model, the wtEGFR expression was strongly associated with the amplification status of the gene. Importantly, activation (phosphorylation) of EGFR was observed in tumors with genomic *EGFR* amplification, while tumors without amplification lacked EGFR expression and activation (Fig. 2 a). To investigate whether there was a difference in major downstream signaling pathways of both phenotypes, we performed western blotting of pAkt, pMAPK and pStat3. These pathways were activated in both phenotypes (Fig. S3), suggesting that common genomic changes independent of EGFR are sufficient drivers of major downstream signaling events.

**Table 1:** Genomic and histological profile of xenografts derived from patient biopsies

Patients	Chromosomal losses	Chromosomal gains	*EGFR* amplification	EGFRvIII status	Histology
A3	*	*	+	+	Highly invasive, non-angiogenic
A5	9p, 10		+	*	
P6	*	*	+	–	
P8	6q, 9p, 10, 13q, 14p, 18q	7, 8q	+	+	
P17	9p, 10, 13	7, 19	+	+	
P22	*	*	+	+	
P1		7	–	–	Angiogenic, less invasive
P2	10, 15p		–	–	
P3	9, 10	7, 21	–	–	
P7	9p, 10, 14, 6q		–	–	
P13	10	7	–	–	
A1	9p, 10, 11q	7	–	*	

* Not determined

We have previously shown that non-angiogenic tumors can spontaneously switch to an angiogenic phenotype upon in vivo passaging [53]. To determine whether wtEGFR amplification, expression, and activation were changed during the angiogenic switch, we analyzed four *EGFR*-amplified, non-angiogenic tumors by serial in vivo passaging. We observed that two tumors switched to an angiogenic phenotype, while the other two maintained a stable invasive phenotype (Fig. 2c). Both phenotypes harbored *EGFR* amplification; however, while the stably invasive tumors showed high levels of wtEGFR expression and phosphorylation (pEGFR), both proteins were downregulated in the core of tumors that switched to an angiogenic phenotype (Fig. 2c). In addition to the wtEGFR protein, various mutants exist, such as EGFRvIII [42]. However, EGFRvIII is expressed in fewer tumor cells than wtEGFR in most patient samples and amplification of *EGFRvIII* occurs to a much lesser extent compared to wt*EGFR* [4]. By evaluating EGFRvIII in our xenografts, we found its expression only in tumors with wt*EGFR* amplification (Fig. S4; Table 1), suggesting a strong association between wt*EGFR* amplification and EGFRvIII expression which has also been described in patient biopsies [57]. Interestingly, EGFRvIII was lost in stably invasive tumors after serial in vivo passages while it was expressed in xenografts that switched to an angiogenic phenotype (Fig. 2d, e). This shows opposite regulations of wtEGFR and EGFRvIII in our xenograft system and indicates that wtEGFR expression is necessary to drive the invasive phenotype, while EGFRvIII is dispensable and might be involved in stimulating angiogenic tumor growth when wtEGFR expression is lost. The relevance of EGFRvIII for tumor angiogenesis has been previously described [6, 11, 36].

### Activation of EGFR in patient samples correlates with invasive/non-angiogenic tumor growth

**Fig. 3:**
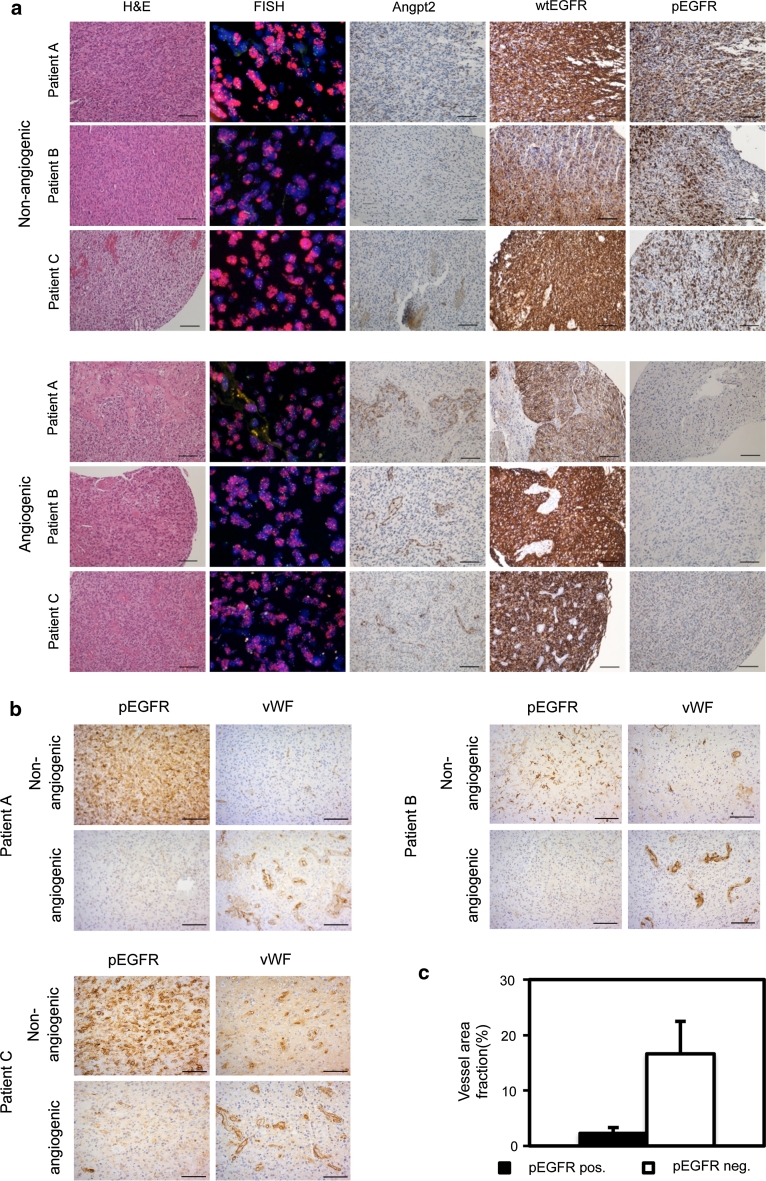
EGFR activation promotes invasive/non-angiogenic tumor growth in GBM patient biopsies. Tissue microarray (TMA) of GBM biopsies. **a** *EGFR*-amplified GBM biopsies as verified by FISH with an *EGFR*/chromosome 7 probe in red and green, respectively. H&E sections and angiopoietin2 stainings indicate non-angiogenic (*upper panel*) versus angiogenic areas (*lower panel*) in *EGFR*-amplified tumors. High pEGFR expression is only found in non-angiogenic areas (*upper panel*). **b** Immunohistochemical staining of pEGFR positive biopsies selected from the TMA with antibodies against pEGFR and vWF. pEGFR positive tumor areas are non-/less angiogenic compared to angiogenic, pEGFR negative areas within the same biopsies. *Scale bars* 100 μm. **c** Area fraction of vascular elements immunostained with vWF from pEGFR positive versus angiogenic, pEGFR negative areas from five different patients. Quantification was performed at ×200 magnification. *P* < 0.001; *n* = 10. Values represent mean ± SD

As our animal model might mimic the development and progression of *EGFR*-amplified tumors in patients, we analyzed a tissue microarray from 206 GBM patients for *EGFR* amplification, protein expression and activation (pEGFR). *EGFR* amplification was found in 87 (41.4 %) patients which is in close agreement with previous results [12]. Moderate-to-high wtEGFR expression in a significant fraction (>10 %) of tumor cells (score ≥ 4) was detected in 77 (88.5 %) out of 87 *EGFR*-amplified tumors and in 26 (21.9 %) out of 119 non-amplified tumors (Table S2). Notably, a significant fraction (>10 %) of moderate-to-high pEGFR positive tumor cells (score ≥ 4) was exclusively found in amplified tumors with high EGFR expression (score ≥ 4). However, only 22 (25.3 %) of 87 amplified tumors had this moderate-to-high pEGFR (score ≥ 4), suggesting that activated EGFR is confined to a subpopulation within *EGFR*-amplified tumors (Table S2). Cells with activated EGFR were found in non-angiogenic or invasive areas where angiogenic vessels were rarely detected (Fig. 3a, upper panel). In contrast, angiogenic biopsies from *EGFR*-amplified tumors with abnormal vessels and high angiopoietin-2 levels showed only a few single or no EGFR-activated cells (Fig. 3a, lower panel). The expression of wtEGFR did not show the same association to invasiveness as pEGFR (Fig. 3a) suggesting that the activation of EGFR, rather than absolute levels of EGFR protein, is the most important factor determining non-angiogenic tumor growth. In addition, we analyzed in detail whole biopsy tissues from seven patients that had moderate-to-high pEGFR expression (score ≥ 4). To verify that pEGFR positive areas were non-angiogenic/invasive, we compared these areas to angiogenic areas from the same biopsies. Indeed, pEGFR positive areas had smaller vessels that covered significantly smaller area fractions of the tumor tissue, compared to pEGFR negative, angiogenic areas (Fig. 3b, c, Fig. S5). Thus, the activation of EGFR correlated with non-angiogenic, invasive growth in both the animal model and in patient biopsies.

### Cetuximab inhibits tumor growth and invasion of *EGFR*-amplified xenografts

**Fig. 4:**
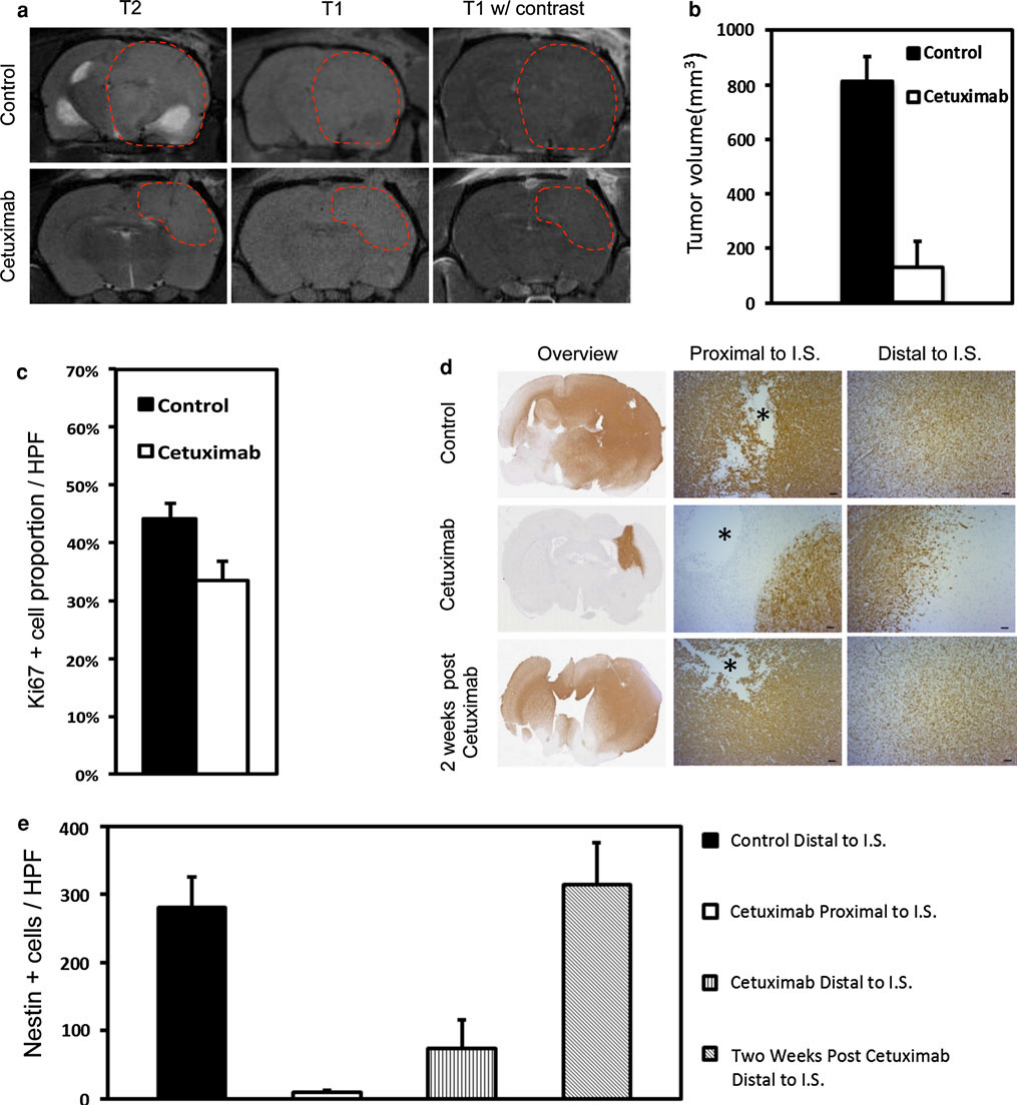
Cetuximab inhibits growth and invasion of *EGFR*-amplified tumors. *EGFR*-amplified xenograft tumors (P8) were treated with cetuximab intracerebrally for 4 weeks using osmotic mini-pumps 6 weeks after tumor implantation. **a** T2- and T1-weighted MRIs of control and treated tumors with and without contrast. Treated tumors are smaller compared to control tumors. **b** Quantification of tumor volumes from MRI pictures. *P* < 0.001, *n* = 3 (controls), *n* = 4 (cetuximab). Values represent mean ± SD. **c** Quantification of proliferating tumor cells from Ki67 immunostained sections at ×400 magnification. *P* < 0.001, *n* = 10. Values represent mean ± SD. **d** Immunohistochemical staining with antibodies against human-specific nestin, used as a tumor cell marker. *Asterisk* marks the injection site (IS). *Scale bars* 100 μm. **e** Quantification of invasive, nestin positive tumor cells. *HPF* high microscopic view field (×400 magnification). *P* < 0.001 except: cetuximab proximal to IS versus cetuximab distal to IS, *P* < 0.01 and control versus 2 weeks post cetuximab, *P* = 0.196; *n* = 10. Values represent mean ± SD

To functionally verify that activation of EGFR is driving invasion in human GBM, we used a clinically relevant anti-EGFR antibody, cetuximab, to block wtEGFR activation in the stably invasive, EGFR-amplified xenografts. To ensure drug delivery to the invasive cells across the BBB, we used a CED technique [22], which leads to a broad distribution of compounds within the brain as described previously [19]. We delivered cetuximab to tumors 6 weeks after tumor implantation using osmotic minipumps. After 4 weeks of continuous cetuximab administration, we observed a dramatic effect on tumor growth and invasion as evidenced by MRI and histology (Fig. 4). The treated tumors were significantly smaller (125.75 mm^3^) compared to control tumors (810 mm^3^) (Fig. 4a, b), which caused neurological symptoms at this stage. Reflecting a slower growth rate, the proliferative activity of treated tumors was significantly lower compared to the controls (Fig. 4c). Importantly, there was a significant block of invasion into the brain in the treatment group, which was most pronounced proximal to the injection site, where tumors showed a demarcated border (Fig. 4d, e). In contrast, the same area in control tumors showed a prominent invasion of tumor cells (Fig. 4d, e). Distal to the injection site, tumor cells in the treatment group showed gradually increased invasiveness, yet infiltration was substantially less compared to control tumors (Fig. 4d, e). This effect might be explained by a lower concentration of cetuximab distal to the injection site. Upon terminating cetuximab administration, the tumors were followed up for two additional weeks. This revealed a reversion to the invasive phenotype similar to what was seen in control tumors (Fig. 4d, e). Thus, these data show that a continuous inhibition of wtEGFR activation is necessary to inhibit GBM invasion.

### Stable inactivation of EGFR by a dominant-negative receptor induces an angiogenic switch in EGFR-amplified xenografts

**Fig. 5:**
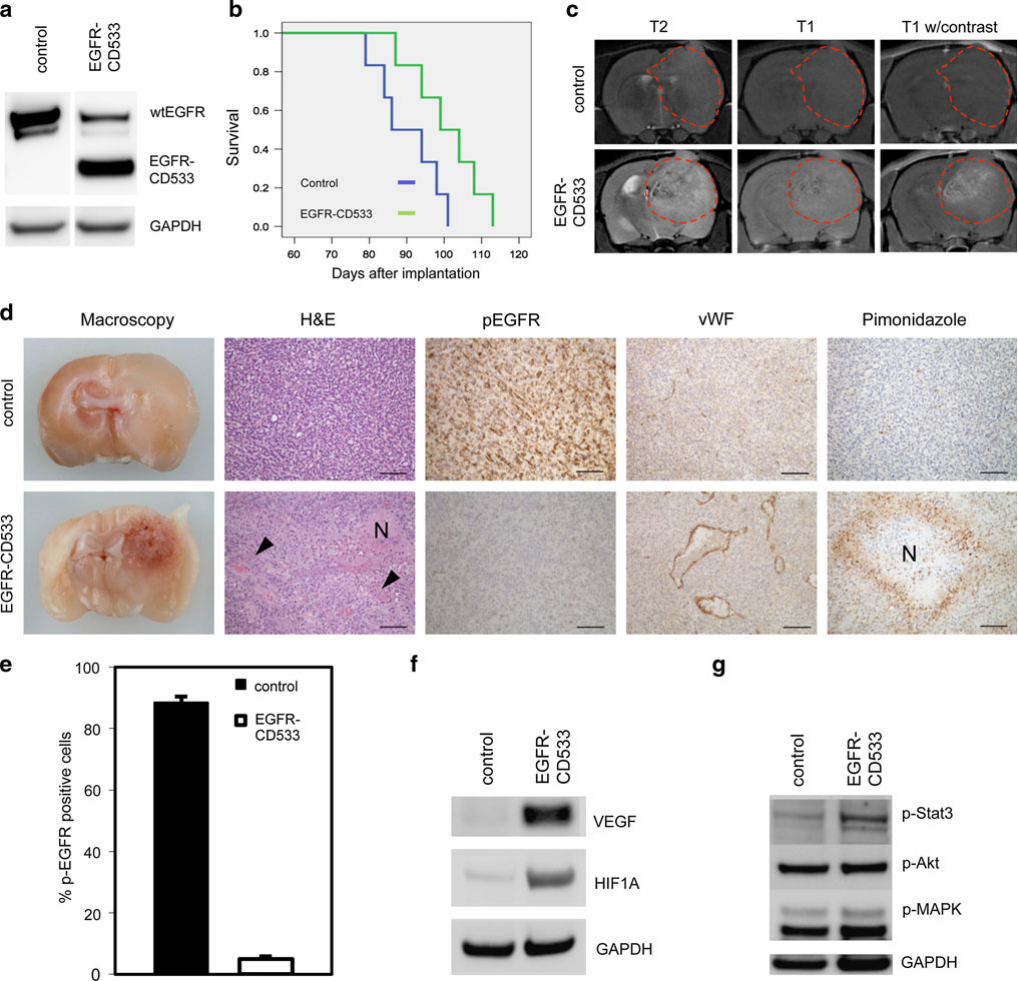
Stable expression of EGFR-CD533 induces an angiogenic switch in *EGFR*-amplified tumors. Tumor spheroids from *EGFR*-amplified tumors (P8) were mock infected or infected with lentiviral vectors carrying EGFR-CD533. Infected spheroids were implanted into the brain of nude rats. **a** Western blot of a control tumor and a tumor transduced with EGFR-CD533 with antibodies against EGFR. **b** Kaplan–Meier survival curve of EGFR-CD533 and control tumors. The difference in survival is statistically significant (log-rank; *P* < 0.05). **c** T2- and T1-weighted MRIs with and without contrast show more demarcated tumors with contrast enhancement in the EGFR-CD533 group, while the control tumors have ill-defined borders and are devoid of contrast enhancement. **d** Macroscopic, coronal view of rat brains with control and EGFR-CD533 tumors. H&E sections show non-angiogenic versus angiogenic tumor growth in control versus EGFR-CD533 tumors. Immunohistochemical staining with antibodies against pEGFR, vWF and pimonidazole, a marker for hypoxia. *N* depicts necrotic areas and the *arrowheads* indicate microvascular proliferation. *Scale bars* 100 μm. **e** Quantification of pEGFR positive cells in tumors from two different animals in each group. Quantification was performed at ×400 magnification. *P* < 0.001; *n* = 10. **f** Western blot with antibodies against HIF1A and VEGF. **g** Western blot with antibodies against pStat3, pAkt and pMAPK

As cetuximab treatment was transient and performed on established tumors, we chose an additional method to stably block wtEGFR activation in the majority of tumor cells through the whole period of tumor development and progression. Using lentiviral vectors, we genetically modified EGFR function by introducing an inactive mutant of the receptor in *EGFR*-amplified tumors. This construct downregulates EGFR signaling in a dominant-negative manner (EGFR-CD533) [13]. Prior to implantation, tumor cells were mock infected, infected with a lentiviral GFP control vector or with a lentiviral EGFR-CD533 construct. Expression of dominant-negative EGFR protein was verified by western blot of tumor tissue (Fig. 5a). Notably, the expression of wtEGFR was strongly downregulated in the dominant-negative group compared to the control tumors (Fig. 5a, Fig. S6).

Kaplan–Meier analysis showed a significantly prolonged survival of animals in the EGFR-CD533 group compared to the control group (Fig. 5b; log-rank: *P* < 0.05), indicating a slower development of the modified tumors. To assess the tumor growth characteristics, we performed MRI. The images revealed circumscribed, contrast-enhancing tumors in the EGFR-CD533 group, while controls showed highly invasive, non-enhancing lesions (Fig. 5c, Fig. S6). The MRI findings were confirmed both at the macroscopic and microscopic level. Angiogenic growth was clearly evident in the EGFR-CD533 tumors, which were circumscribed and harbored microvascular proliferations and necroses. Angiogenesis was absent in the diffusely invasive growing tumors of the control group (Fig. 5d, Fig. S6). Importantly, EGFR activation (pEGFR) was significantly inhibited in the EGFR-CD533 tumors compared to the control tumors (Fig. 5d, e, Fig. S6).

To determine whether the tumors were hypoxic, pimonidazole staining was performed. While hypoxic areas were identified around necroses in the EGFR-CD533 group, no hypoxic regions were observed in control tumors (Fig. 5d). We further determined the expression of HIF1A and VEGFA to verify induction of an angiogenic switch at the molecular level. Upregulation of HIF1A and VEGFA occurred in the EGFR-CD533 transduced, angiogenic tumors whereas both proteins were absent/less expressed in the control tumors (Fig. 5f, Fig. S6). Functional analysis of gene expression data using Ingenuity Pathway Analysis (IPA) showed that the biological function “angiogenesis” was highly enriched (*p* 2.22 × 10^8^) in the dataset. *Z* score analysis revealed that angiogenesis was significantly increased (*z* score 3,305) in EGFR-CD533 tumors (Table S3). Subsequently, we used IPA to query for significantly altered upstream regulators in the dataset. HIF1A was the top enriched upstream regulator (*P* 3.45 × 10^11^; Fig. S7).

To analyze whether inhibition of EGFR activation affected major downstream signaling pathways, we performed western blots for phosphorylated Akt, MAPK and Stat3. While Akt and MAPK activation were not changed, phosphorylation of Stat3 was enhanced in the EGFR-CD533 group (Fig. 5g). Thus, major downstream signaling was not blocked by inhibiting EGFR activation which is in line with previous clinical studies using EGFR inhibitors [24, 33, 41] and might in part explain the resistance to anti-EGFR therapy.

### Stable inactivation of EGFR induces a mesenchymal to epithelial-like transition in EGFR-amplified xenografts

**Fig. 6:**
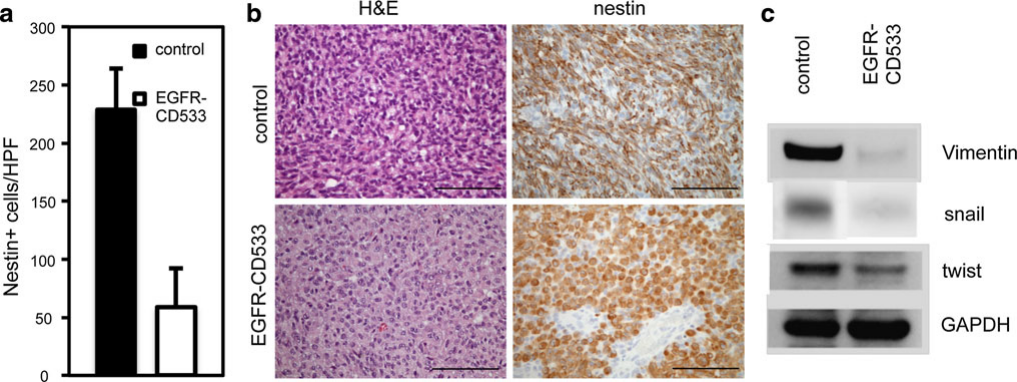
Expression of EGFR-CD533 promotes a mesenchymal to epithelial-like transition in *EGFR*-amplified tumors. **a** Quantification of invasive cells in cortical areas from two different animals in each group. *HPF* high microscopic view field (×400 magnification). *Asterisk* *P* < 0.001; *n* = 10. Values represent mean ± SD. **b** Hematoxylin and eosin (H&E) and immunohistochemical staining against nestin show mesenchymal shape of cells in control versus epithelial-like shape in EGFR-CD533 tumors. *Scale bars* 100 μm. **c** Western blot with antibodies against vimentin, snail and twist

To verify that inhibition of EGFR activity affected the invasiveness of tumor cells, we performed detailed cell counts, which revealed less invasive cells in cortical areas within the EGFR-CD533 group compared to the control group (Fig. 6a, Fig. S6). Loss of invasiveness and cell motility is often accompanied by changes in morphology, also referred to as mesenchymal to epithelial transition (MET) [26]. In the EGFR-CD533 group, histology and nestin staining showed tumor cells with an epithelial-like phenotype compared to mesenchymal control cells (Fig. 6 b). MET was confirmed at the molecular level by analyzing vimentin, snail and twist expression, which are established markers for mesenchymal tumor cells and are upregulated upon epithelial to mesenchymal transition as well as during the metastatic process of epithelial cancers [8, 61]. Vimentin, snail and twist were all strongly downregulated in EGFR-CD533 tumors as compared to controls (Fig. 6c).

## Discussion

In summary, our data show that tumor cell invasion is strongly associated with wtEGFR amplification and activation and that this process is independent of angiogenesis in human GBMs. The selection for *EGFR* amplification in our animal model, the coincidence of wtEGFR expression and invasion as an early process in tumorigenesis, and the stem-like properties of these cells described previously [53] suggest a functional role for wtEGFR in cancer development. This is supported by a recent study, demonstrating the importance of wtEGFR for tumor development and invasive growth within a stem-like human GBM xenograft model [40]. Taken together, this study and our results show that the non-angiogenic wtEGFR-amplified population clearly represents a different subset of cancer cells as compared to the highly angiogenic cancer stem-like cells, which have been proposed to be the only tumorigenic cells within GBMs [2, 20, 32, 55].

Although the inhibition of EGFR activation significantly reduced tumor growth and invasion in our model, tumor cells had the capacity to induce an angiogenic program and thereby escape the invasion block as shown by stable expression of dominant-negative EGFR. In this experimental set-up, we observed a mesenchymal to epithelial-like transition, which might explain the inability of EGFR-CD533 expressing tumor cells to escape from hypoxic areas through invasion and instead induce an angiogenic program by upregulating HIF1A. This was verified by a microarray analysis showing that genes which are transcriptionally activated by HIF1A were upregulated in the EGFR-CD533 expressing tumors. Recently, Lu et al. [35] observed that c-Met induced an epithelial to mesenchymal transition and invasive phenotype after VEGF inhibition in glioblastoma. This indicates that in addition to EGFR other tyrosine kinase receptors might be important for invasion and a mesenchymal phenotype in high-grade glioma. In contrast, low grade gliomas often do not show amplification of tyrosine kinase receptors [16], but are also invasive. Thus, in these tumor types other mechanisms driving tumor invasion might be responsible.

Our results highlight the dynamic nature of highly malignant tumor cells that have a number of genetic changes in common such as 9p and 10q deletions that disrupt PTEN, p53 and RB tumor suppressor pathways [9, 49]. The inactivation of these pathways is probably sufficient to drive tumor growth as also verified in genetic mouse models of GBMs [1, 62]. Inactivation of these tumor suppressor genes may also activate major downstream signaling events such as AKT, MAPK and Stat3 which, as shown in the present study, is not dependent on wtEGFR activation; however, the EGFR status as demonstrated here has an important impact on the balance between invasive and angiogenic tumor growth. In particular, patient tumors are highly heterogenous and contain both, cells with and without *EGFR* amplification. Cells with high *EGFR* amplification are more frequent in invasive areas as compared to the main angiogenic tumor mass [48, 54]. Additional support has been provided in a recent study showing that the invasive areas of GBMs with co-amplification of *EGFR* and *PDGFR* exclusively contain *EGFR*-amplified cells, while *PDGFR* amplified cells are only found in the main tumor mass [54]. Accordingly, the angiogenic switch in human tumors might be induced by less migratory cells in which EGFR signaling is absent/low. In this context, we showed that only a subset of *EGFR*-amplified tumor cells within GBM biopsies had a highly activated EGFR and importantly, these cells were found in non-angiogenic/invasive areas.

In our xenograft model system, wtEGFR was the main driver of invasion, whereas mutated EGFRvIII was lost after serial passaging. In contrast, wtEGFR was downregulated in tumors that switched to an angiogenic phenotype, while EGFRvIII was stably expressed. This shows opposite regulations of wtEGFR versus EGFRvIII in our xenograft system and strong associations to either invasion or angiogenesis, respectively. Several studies have shown that EGFRvIII is responsible for angiogenic growth within GBM animal models and cell lines using overexpression approaches [6, 11, 28, 36] and that EGFRvIII differs from wtEGFR signaling [29, 44–46]. Although it has been demonstrated that wtEGFR also can induce upregulation of angiogenic factors in glioma cell lines in vitro [21, 28, 37, 52], there is lack of evidence that this can be a mechanism in vivo. In contrast, by preserving naturally occuring *EGFR*-amplified cells in vivo, we have clearly shown that wtEGFR is a driver of invasion in human GBM in vivo. The mechanism of how the gene expression of EGFRvIII and wtEGFR are regulated in our animal model and also in human GBM still needs to be identified. However, a recent study suggests that at least EGFRvIII is epigenetically regulated [15].

The EGFR-activated tumor subpopulation is an important target for therapy as these cells are highly invasive and, accordingly, have the capacity to escape current therapies. In addition, an intact BBB inherently impedes drug delivery to invasive and non-angiogenic tumor regions. In our xenograft model, we clearly demonstrate that local delivery of an anti-EGFR antibody significantly inhibits tumor growth and invasion. These results highlight the importance of anti-EGFR therapy as an anti-invasive treatment strategy for GBM and most likely also explain why systemic administration of otherwise effective anti-EGFR drugs fails to show substantial effects in the clinic [41]. Although it has been postulated that small molecule inhibitors may successfully circumvent the drug penetration problem often associated with antibody therapies involving the CNS, recent observations show that drug transporters in endothelial cells of intact vessels prevent effective penetration of these molecules [17, 31, 38]. Thus, a major challenge in targeting these invasive, EGFR-activated tumor subpopulations will be to effectively deliver bioactive molecules across the BBB and at the same time inhibit potential angiogenic escape mechanisms.

## Electronic supplementary material

Below is the link to the electronic supplementary material.

Supplementary Table S1 (DOCX 39 kb)

Supplementary Table S2 (DOC 31 kb)

Supplementary Table S3 (DOCX 121 kb)

**Figure S1 Two different phenotypes of human glioblastoma xenografts**. The invasive phenotype (upper panel) shows no signs of angiogenesis and diffuse invasion into the brain. vWF staining demonstrates vessels with a normal endothelium. The angiogenic phenotype (lower panel) shows microvascular proliferation (arrowheads) and necroses (N) and less invasive growth at the border. vWF staining shows microvascular proliferation (xenograft A1) and dilated macrovessels (xenograft P1). The xenografts are derived from different patients (A3, P8, A1 and P1), which are further characterized in table 1. Scale bars 100μm (TIFF 1,436 kb)

**Figure S2 Expression of stem cell markers in invasive versus angiogenic xenografts.** (**a**) Immunhistochemical staing of sections from invasive (P17) and angiogenic tumors (P3) with antibodies against nestin and sox2. Both phenotypes show strong expression of nestin and sox2. Scale bars 100μm (**b**) western blot of 3 invasive (P6, P8, P22) and 3 angiogenic xenografts (P1, P3, P13) with antibodies against CD133 (TIFF 3,156 kb)

**Figure S3. Major downstream signaling in invasive versus angiogenic xenografts.** Western blots with antibodies against phospho-Akt, -MAPK and -Stat3. The pathways are active in both, invasive (P6, P8, P22) and angiogenic (P1, P3, P13) phenotypes (TIFF 224 kb)

**Figure S4 Expression of EGFRvIII in invasive versus angiogenic xenografts.** EGFRvIII western blot of 3 invasive (P6, P8, P22) and 3 angiogenic (P1, P3, P13) low passage xenografts (TIFF 126 kb)

**Figure S5 High levels of EGFR phosphorylation are detected in non-angiogenic areas of patient biopsies with EGFR amplification.** Immunohistochemical staining of pEGFR positive biopsies taken from the TMA with antibodies against pEGFR and vWF. pEGFR positive tumor areas are non-/less angiogenic compared to angiogenic, pEGFR negative areas within the same biopsies (TIFF 5,480 kb)

**Figure S6 Mock-infected control tumors show no difference in invasive and angiogenic properties compared to lentiviral GFP-infected tumors.** Tumor spheroids from *EGFR* amplified tumors were mock-infected or infected with lentiviral control vectors carrying GFP. Infected spheroids were implanted into the brain of nude rats. **(a)** Western blot of a control tumor and a tumor transduced with GFP with antibodies against EGFR. **(b)** T2- and T1-weighted MRIs with and without contrast show invasive tumors without contrast enhancement in both groups. **(c)** H&E sections show invasive tumor growth in both groups. Immunhistochemical staining with antibodies against GFP, pEGFR, and vWF Scale bars, 50μm. **(d)** Quantification of pEGFR positive cells in tumors from one animal in each group. Quantification was performed at 400x magnification. P<0.001; n=5. **(e)** Western blot with antibodies against HIF1A and VEGF. Tumors transduced with EGFR-CD533 were used as positive control. **(f)** Quantification of invasive cells in cortical areas from two different animals in each group. ’HPF’, high microscopic view field (400x magnification). P<0.001; n=10. Values represent mean ± s.d. (TIFF 4,223 kb)

**Figure S7 HIF1A is a key upstream regulator of angiogenesis-related genes in EGFR-CD533 tumors.** Graphical representation after functional analysis using Ingenuity Pathway Analysis. Genes regulated by HIF1A, which are altered in the dataset are represented. The activation state of HIF1A is inferred from the expression values of its downstream target genes. See prediction legend in the inset for the details of the relationships. (TIFF 2,022 kb)
