# Supplementary material for: EGFR wild-type amplification and activation promote invasion and development of glioblastoma independent of angiogenesis
Source: Acta Neuropathol. 2013 Feb 22;125(5):683–98. doi: 10.1007/s00401-013-1101-1 (PMC3631314; doi:10.1007/s00401-013-1101-1)
Supplement: Supplementary file 1 — Supplementary Table S1 (DOCX 39 kb) [file 401_2013_1101_MOESM1_ESM.docx]

Table S1

**Angiogenic features of xenografts derived from patient biopsies**

| patients | Dilatated macrovessels | Endothelial hyperplasia | Microvascular proliferation | Necroses |
| --- | --- | --- | --- | --- |
| P1 | + | + | + | + |
| P2 | + | + | - | - |
| P3 | + | + | (+) | (+) |
| P7 | + | + | - | + |
| P13 | + | + | + | + |
| A1 | + | + | + | + |

(+) initially not present, but after in vivo passaging
