# Supplementary material for: EGFR wild-type amplification and activation promote invasion and development of glioblastoma independent of angiogenesis
Source: Acta Neuropathol. 2013 Feb 22;125(5):683–98. doi: 10.1007/s00401-013-1101-1 (PMC3631314; doi:10.1007/s00401-013-1101-1)
Supplement: Supplementary file 2 — Supplementary Table S2 (DOC 31 kb) [file 401_2013_1101_MOESM2_ESM.doc]

Table S2

**wtEGFR and pEGFR expression in GBM**

| EGFR status | Score* | wtEGFR (%) | pEGFR (%) |
| --- | --- | --- | --- |
| amplified | 0 | 3 (3.4) | 27 (31) |
| 87 (42.2%) | <4 | 7 (8.1) | 38 (43.7) |
|  | ≥4 | **77 (88.5)** | **22** **(25.3)** |
| Non-amplified | 0 | 65 (54.6) | 104 (87.4) |
| 119 (57.8%) | <4 | 28 (23.5) | 15 (12.6) |
|  | ≥4 | **26 (21.9)** | **0 (0)** |

* Expression Score was assessed independently by two observers. The detailed scoring scheme is described in materials and methods.
