# Supplementary material for: EGFR wild-type amplification and activation promote invasion and development of glioblastoma independent of angiogenesis
Source: Acta Neuropathol. 2013 Feb 22;125(5):683–98. doi: 10.1007/s00401-013-1101-1 (PMC3631314; doi:10.1007/s00401-013-1101-1)
Supplement: Supplementary file 3 — Supplementary Table S3 (DOCX 121 kb) [file 401_2013_1101_MOESM3_ESM.docx]

Table S3: List of genes associated with the biological function angiogenesis and their regulations in EGFR-CD533 compared to control tumors.

| Genes in dataset | Prediction | Log Ratio | Findings |
| --- | --- | --- | --- |
| ECM1 | Increased | 1.044 | Increases (3) |
| NPPA | Increased | -0.950 | Decreases (1) |
| ADM | Increased | 1.737 | Increases (3) |
| DGKA | Increased | 0.659 | Increases (1) |
| ANGPTL4 | Increased | 2.711 | Increases (2) |
| IL6 | Increased | 1.305 | Increases (1) |
| ITGB3 | Increased | 2.413 | Increases (2) |
| EFNB2 | Increased | 2.671 | Increases (1) |
| EGF | Increased | 1.480 | Increases (2) |
| S1PR3 | Increased | 0.454 | Increases (5) |
| FOXO3 | Increased | -0.674 | Decreases (1) |
| LGALS3 | Increased | 2.629 | Increases (1) |
| ALCAM | Increased | -1.492 | Decreases (1) |
| RGCC | Increased | -1.318 | Decreases (1) |
| TEK | Increased | 0.878 | Increases (3) |
| FGF2 | Increased | 0.467 | Increases (20) |
| CXCL1 | Increased | 0.121 | Increases (4) |
| ANGPT2 | Increased | 1.226 | Increases (4) |
| CTSB | Increased | 0.896 | Increases (1) |
| VASH1 | Increased | -0.669 | Decreases (4) |
| CAV1 | Increased | 4.368 | Increases (1) |
| MET | Increased | 2.818 | Increases (1) |
| HOXB3 | Increased | 0.958 | Increases (2) |
| PLAUR | Increased | 2.278 | Increases (2) |
| F3 | Increased | 0.935 | Increases (1) |
| TNC | Increased | 1.167 | Increases (2) |
| mir-10 | Increased | -0.255 | Decreases (4) |
| SERPINE1 | Increased | 3.539 | Increases (2) |
| HOXD10 | Increased | -0.760 | Decreases (1) |
| PLD1 | Increased | 2.243 | Increases (2) |
| CCL2 | Increased | 2.978 | Increases (3) |
| WNT5A | Increased | 1.664 | Increases (1) |
| MMP9 | Increased | 0.420 | Increases (1) |
| VEGFA | Increased | 2.531 | Increases (43) |

Log ratio is the observed value in EGFR-CD533 versus control tumors

Numbers in brackets indicate published evidences indexed in Ingenuity Pathway Analysis

Coloured rows indicate decreased expression leading to an increase in function (negative regulators)
