# Supplementary figures and images for: EGFR wild-type amplification and activation promote invasion and development of glioblastoma independent of angiogenesis
Source: Acta Neuropathol. 2013 Feb 22;125(5):683–98. doi: 10.1007/s00401-013-1101-1 (PMC3631314; doi:10.1007/s00401-013-1101-1)

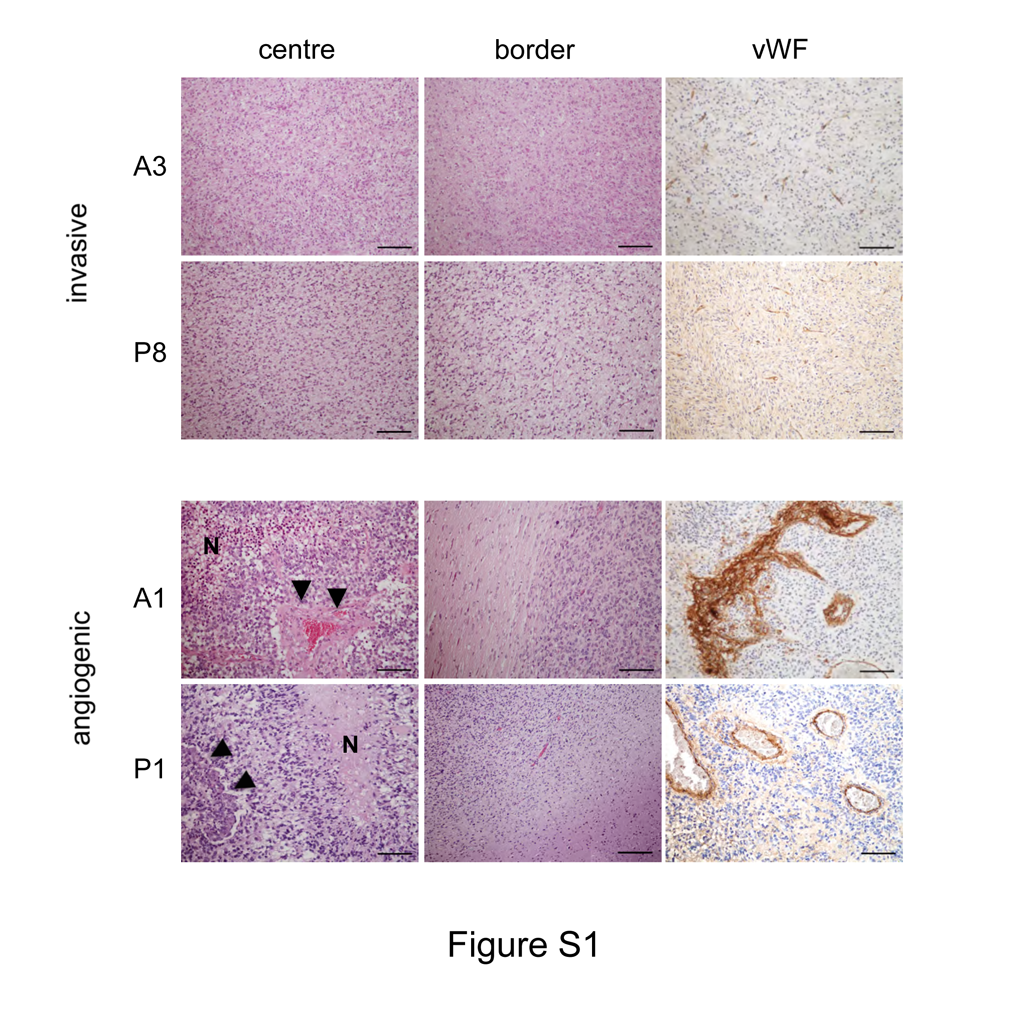

Supplement: Supplementary file 4 — Figure S1 Two different phenotypes of human glioblastoma xenografts. The invasive phenotype (upper panel) shows no signs of angiogenesis and diffuse invasion into the brain. vWF staining demonstrates vessels with a normal endothelium. The angiogenic phenotype (lower panel) shows microvascular proliferation (arrowheads) and necroses (N) and less invasive growth at the border. vWF staining shows microvascular proliferation (xenograft A1) and dilated macrovessels (xenograft P1). The xenografts are derived from different patients (A3, P8, A1 and P1), which are further characterized in table 1. Scale bars 100μm (TIFF 1,436 kb) [file 401_2013_1101_MOESM4_ESM.tif]

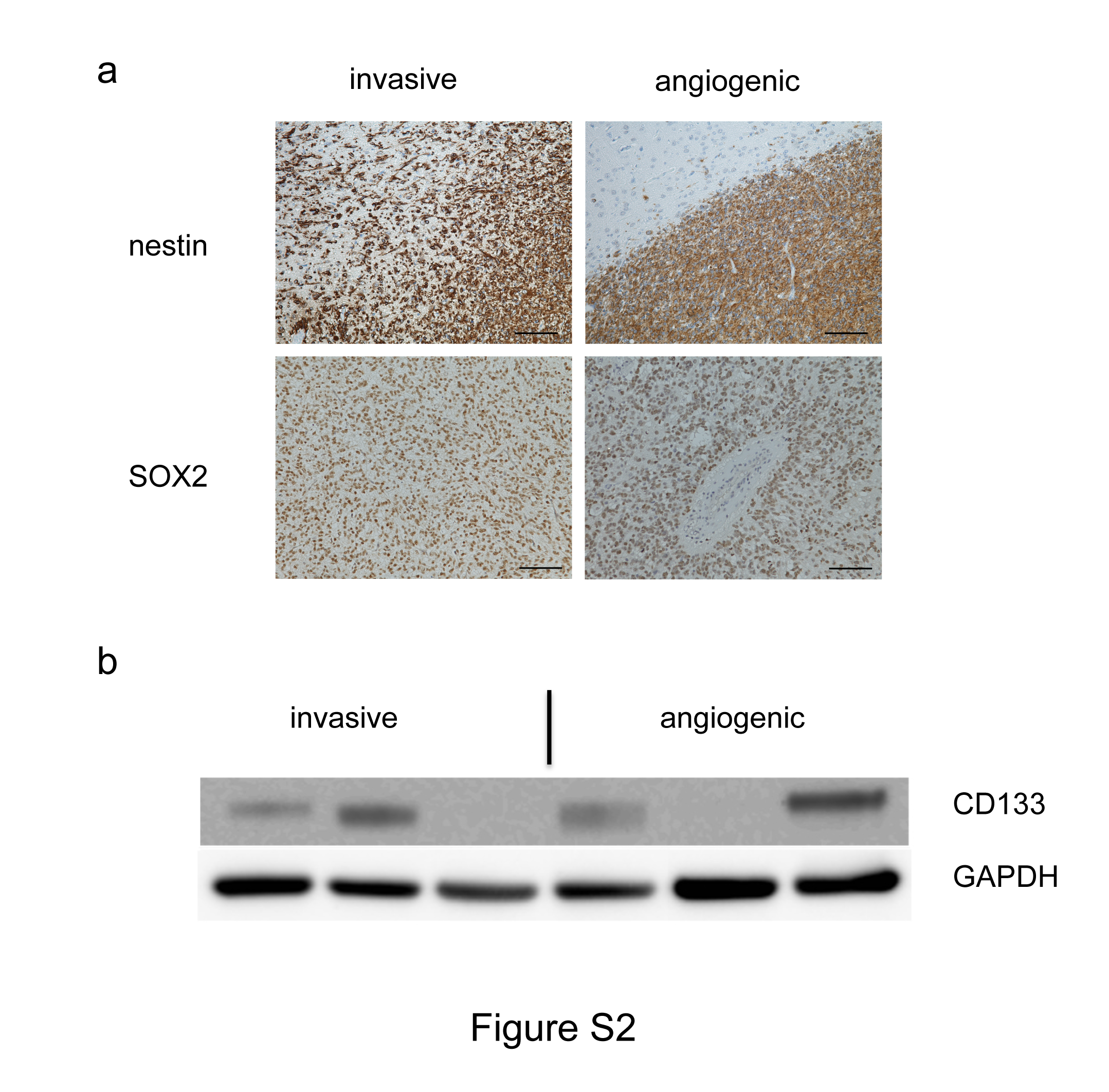

Supplement: Supplementary file 5 — Figure S2 Expression of stem cell markers in invasive versus angiogenic xenografts. (a) Immunhistochemical staing of sections from invasive (P17) and angiogenic tumors (P3) with antibodies against nestin and sox2. Both phenotypes show strong expression of nestin and sox2. Scale bars 100μm (b) western blot of 3 invasive (P6, P8, P22) and 3 angiogenic xenografts (P1, P3, P13) with antibodies against CD133 (TIFF 3,156 kb) [file 401_2013_1101_MOESM5_ESM.tif]

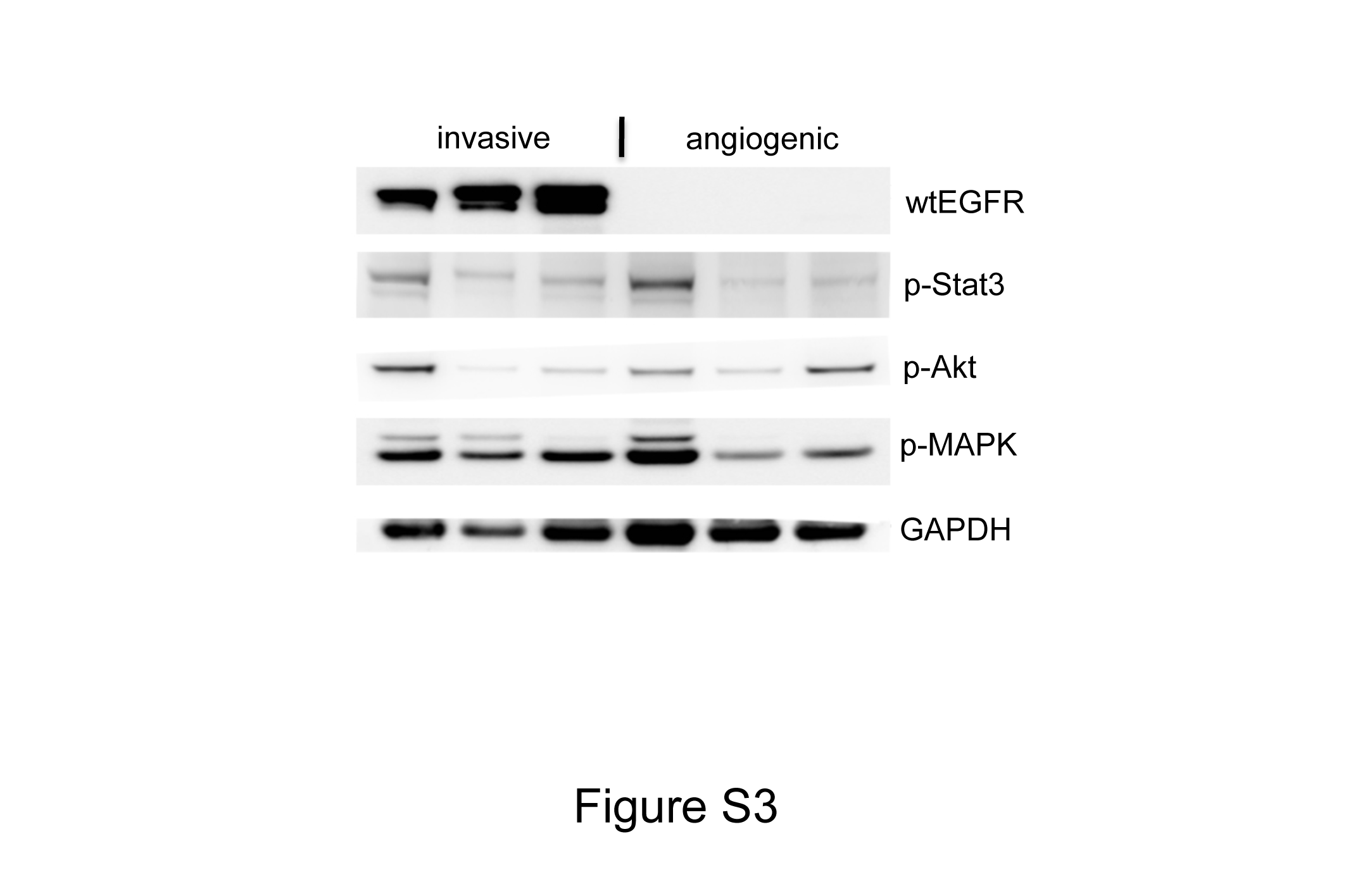

Supplement: Supplementary file 6 — Figure S3. Major downstream signaling in invasive versus angiogenic xenografts. Western blots with antibodies against phospho-Akt, -MAPK and -Stat3. The pathways are active in both, invasive (P6, P8, P22) and angiogenic (P1, P3, P13) phenotypes (TIFF 224 kb) [file 401_2013_1101_MOESM6_ESM.tif]

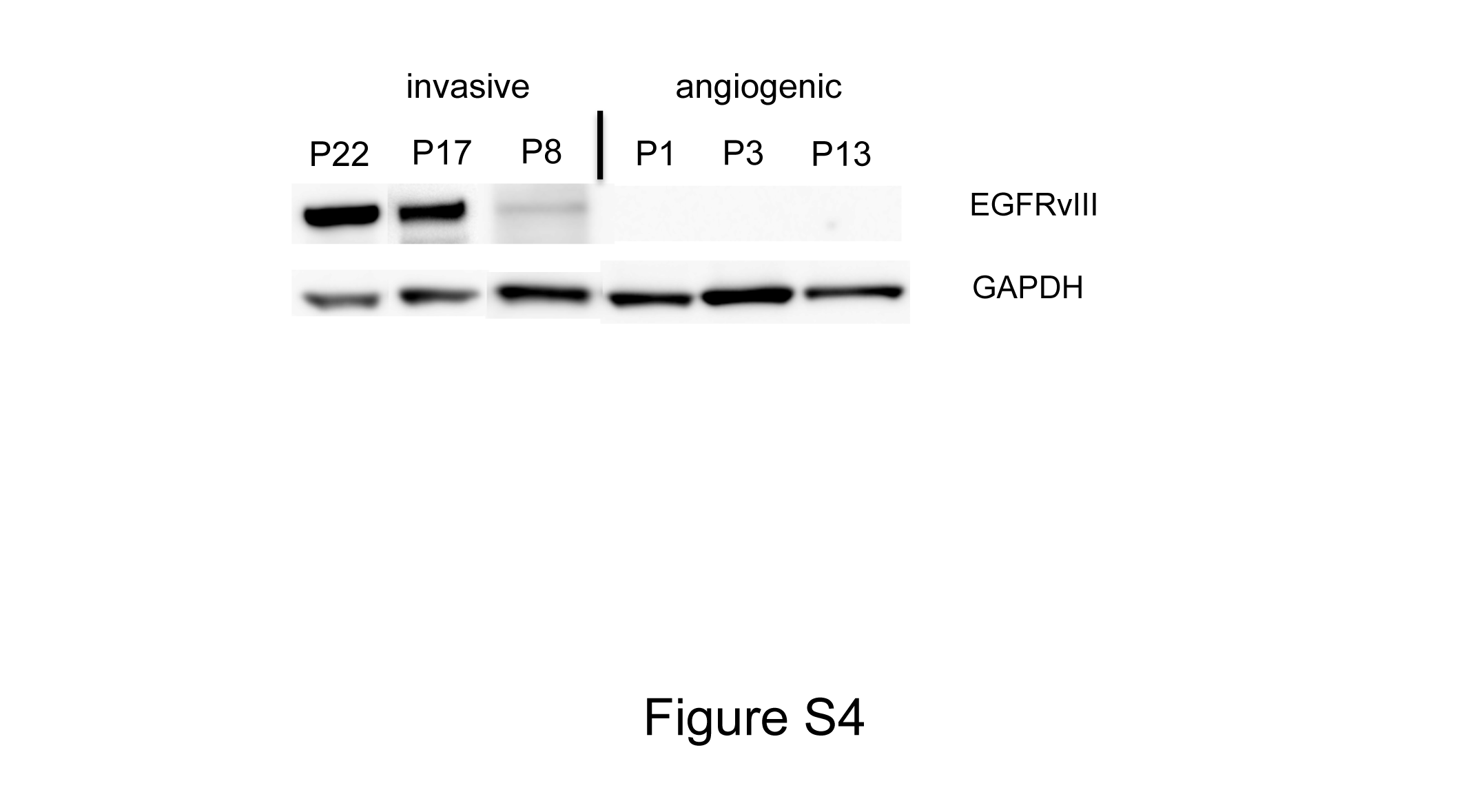

Supplement: Supplementary file 7 — Figure S4 Expression of EGFRvIII in invasive versus angiogenic xenografts. EGFRvIII western blot of 3 invasive (P6, P8, P22) and 3 angiogenic (P1, P3, P13) low passage xenografts (TIFF 126 kb) [file 401_2013_1101_MOESM7_ESM.tif]

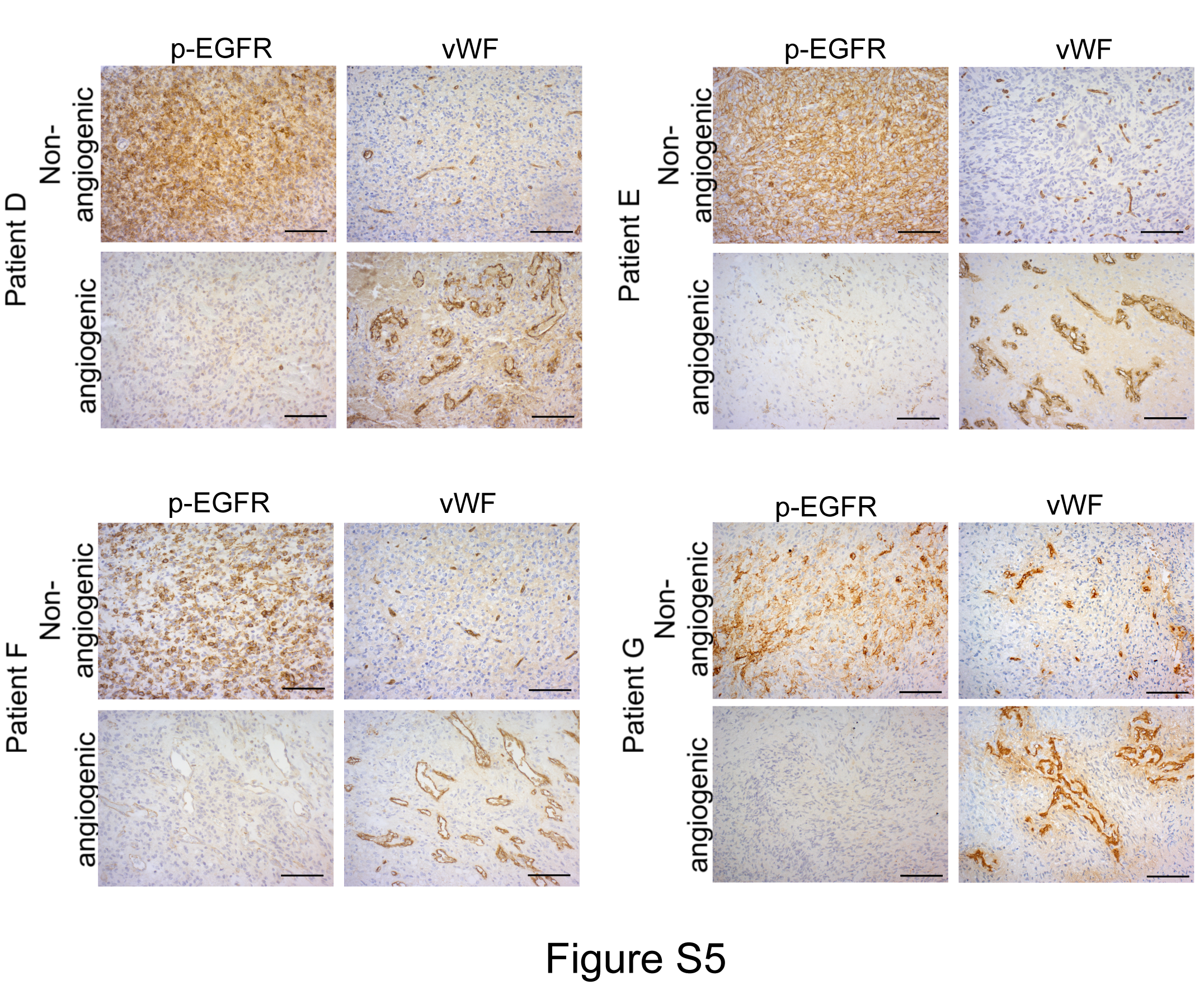

Supplement: Supplementary file 8 — Figure S5 High levels of EGFR phosphorylation are detected in non-angiogenic areas of patient biopsies with EGFR amplification. Immunohistochemical staining of pEGFR positive biopsies taken from the TMA with antibodies against pEGFR and vWF. pEGFR positive tumor areas are non-/less angiogenic compared to angiogenic, pEGFR negative areas within the same biopsies (TIFF 5,480 kb) [file 401_2013_1101_MOESM8_ESM.tif]

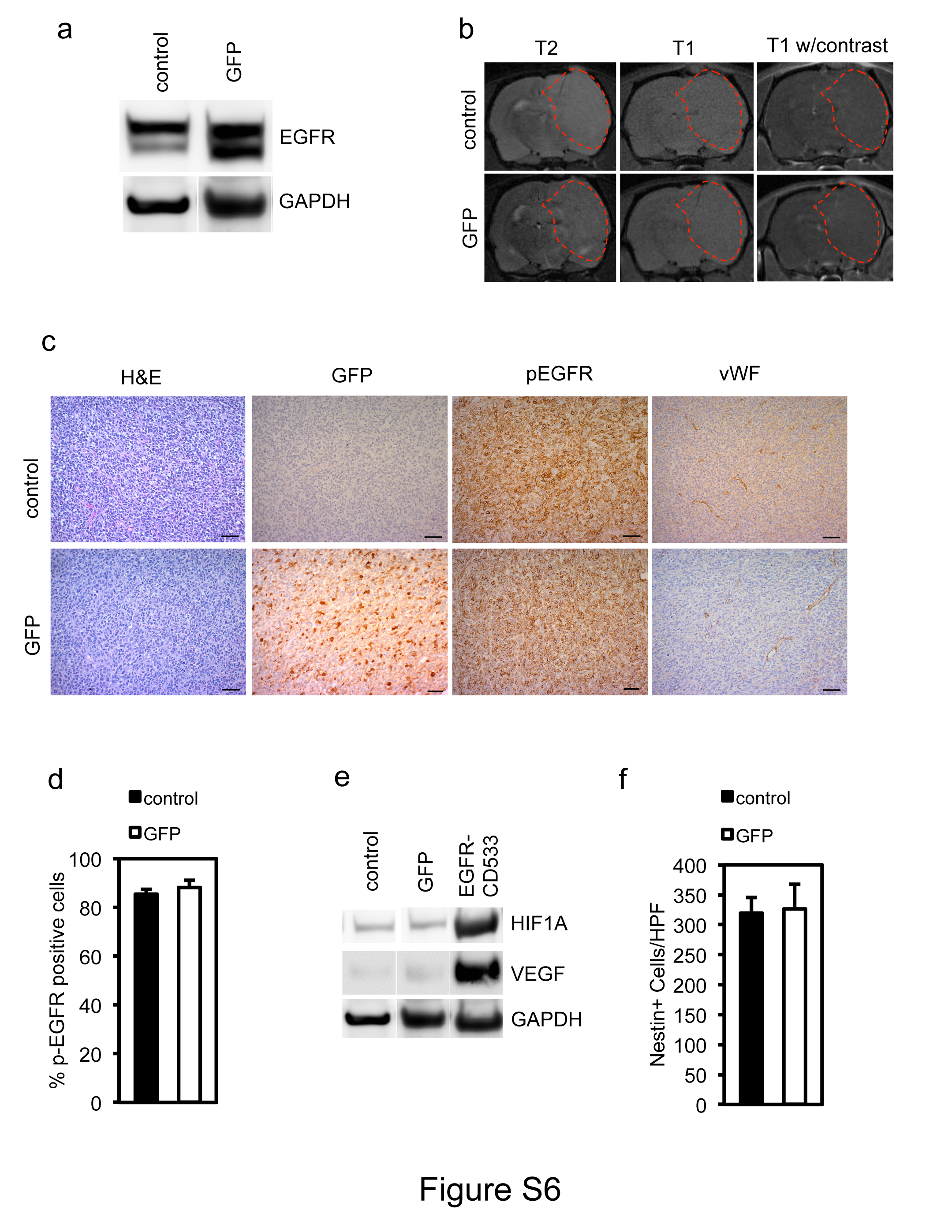

Supplement: Supplementary file 9 — Figure S6 Mock-infected control tumors show no difference in invasive and angiogenic properties compared to lentiviral GFP-infected tumors. Tumor spheroids from EGFR amplified tumors were mock-infected or infected with lentiviral control vectors carrying GFP. Infected spheroids were implanted into the brain of nude rats. (a) Western blot of a control tumor and a tumor transduced with GFP with antibodies against EGFR. (b) T2- and T1-weighted MRIs with and without contrast show invasive tumors without contrast enhancement in both groups. (c) H&E sections show invasive tumor growth in both groups. Immunhistochemical staining with antibodies against GFP, pEGFR, and vWF Scale bars, 50μm. (d) Quantification of pEGFR positive cells in tumors from one animal in each group. Quantification was performed at 400x magnification. P<0.001; n=5. (e) Western blot with antibodies against HIF1A and VEGF. Tumors transduced with EGFR-CD533 were used as positive control. (f) Quantification of invasive cells in cortical areas from two different animals in each group. ’HPF’, high microscopic view field (400x magnification). P<0.001; n=10. Values represent mean ± s.d. (TIFF 4,223 kb) [file 401_2013_1101_MOESM9_ESM.tif]

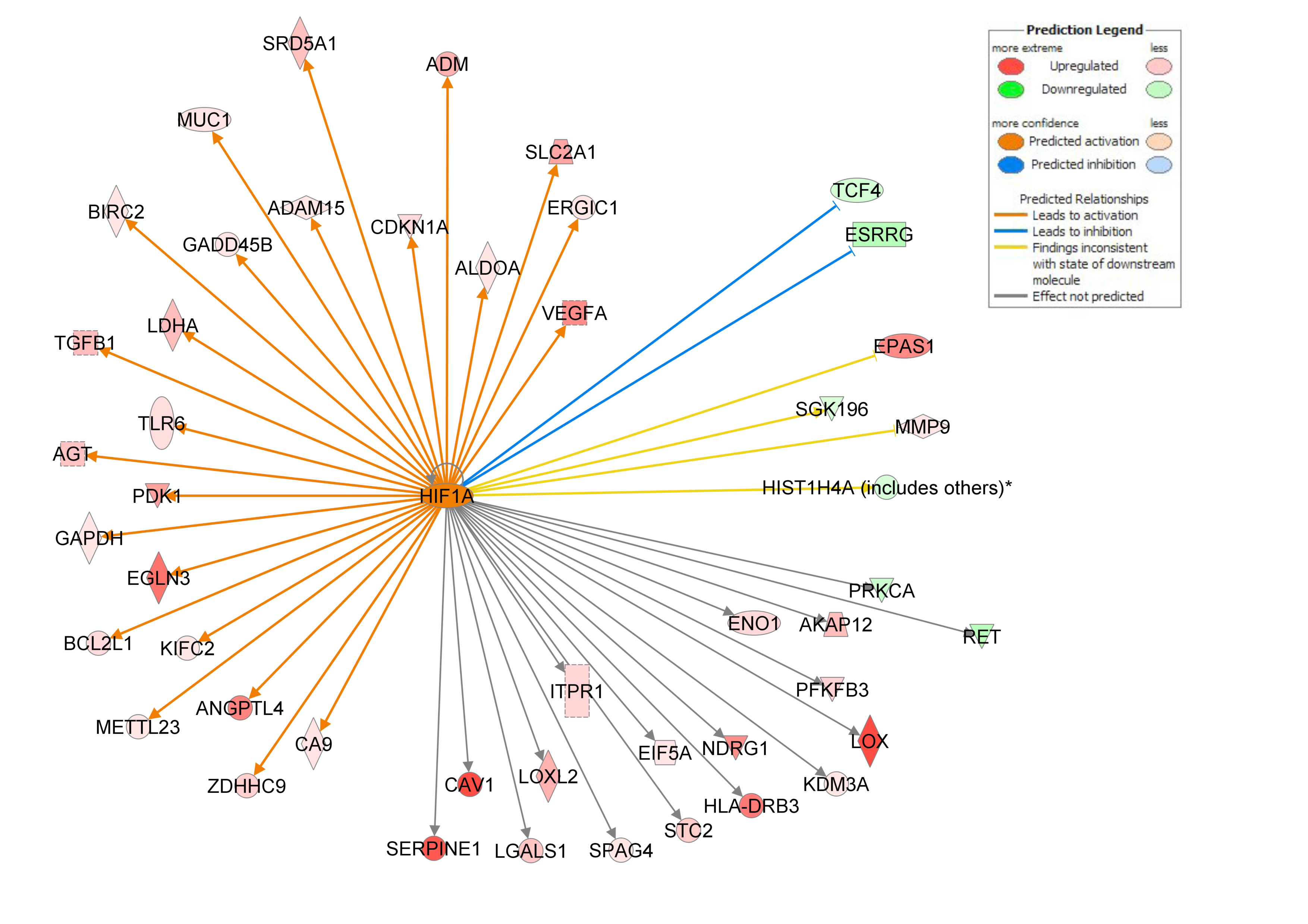

Supplement: Supplementary file 10 — Figure S7 HIF1A is a key upstream regulator of angiogenesis-related genes in EGFR-CD533 tumors. Graphical representation after functional analysis using Ingenuity Pathway Analysis. Genes regulated by HIF1A, which are altered in the dataset are represented. The activation state of HIF1A is inferred from the expression values of its downstream target genes. See prediction legend in the inset for the details of the relationships. (TIFF 2,022 kb) [file 401_2013_1101_MOESM10_ESM.tif]
